# Investigating the causal relationship between gut microbiota and gastroenteropancreatic neuroendocrine neoplasms: a bidirectional Mendelian randomization study

**DOI:** 10.3389/fmicb.2024.1420167

**Published:** 2024-08-13

**Authors:** Chun-yu Zhang, Shi-jing Jiang, Jing-jing Cao, Yan Xu, Xiao-yu Wang, Rui Li, Zhi-wei Miao

**Affiliations:** ^1^Zhangjiagang Hospital of Traditional Chinese Medicine, Nanjing University of Chinese Medicine, Nanjing, China; ^2^Nanjing University of Chinese Medicine, Nanjing, China; ^3^The First Affiliated Hospital of Soochow University, Suzhou, China

**Keywords:** gut microbiota, gastroenteropancreatic neuroendocrine neoplasms, Mendelian randomization, causality, genome wide association study

## Abstract

**Background:**

The interaction between the intestinal flora and gastroenteropancreatic neuroendocrine neoplasms (GEP-NENs) remains poorly understood, despite the known effect of the gut microbiota on gastrointestinal adenocarcinomas. Hence, the present research aimed to determine the potential causal correlation between the intestinal flora and GEP-NENs by conducting a bidirectional Mendelian randomization (MR) analysis.

**Methods:**

Two-sample MR analysis was conducted using the summary statistics of the gut microbiota from the MiBioGen consortium and those of GEP-NENs from the FinnGen research project. The inverse-variance weighted approach was utilized as the primary analytical method. To enhance the robustness of our findings, multiple sensitivity tests were performed, including Cochran’s Q test for evaluating heterogeneity, the MR-Egger intercept test to detect horizontal pleiotropy, and the MR-PRESSO test to identify outliers and assess pleiotropy bias. Additionally, a leave-one-out analysis was performed to validate the consistency of our findings. The MR-Steiger test was also utilized to determine the causal direction in the correlation between the gut microbiota and GEP-NENs. Finally, a reverse MR analysis was performed to assess reverse causality between the intestinal flora and GEP-NENs.

**Results:**

We identified 42 taxa of the gut microbiota that were potentially causally associated with GEP-NENs; of these taxa, 7, 8, 11, and 16 taxa were causally associated with pancreatic NENs, colorectal NENs, small intestinal NENs, and gastric NENs, respectively. After adjusting for false discovery rate (FDR) correction, we found significant causal links of Euryarchaeota with small intestinal NENs and Family XIII UCG-001 with gastric NENs. The sensitivity analyses confirmed the stability of these correlations. In the reverse MR analysis, colorectal NENs and small intestinal NENs were found to be associated with variations in 8 and 6 different taxa of the gut microbiota, respectively. After adjusting for FDR correction, no significant causal links were detected between GEP-NENs and the intestinal flora.

**Conclusion:**

The present study reveals a potential causal association between certain taxa of the intestinal flora and GEP-NENs, thus providing new perspectives regarding the role of the intestinal flora in the development of these tumors. These insights could provide innovative approaches to screen and prevent these diseases.

## 1 Introduction

NENs constitute a heterogeneous group of tumors originating from neuroendocrine cells localized in various organs and systems in the body, particularly in the gastroenteropancreatic (GEP) tract (Modica et al., 2024a,b). The colorectum (43.9%), small intestine (30.8%), pancreas (12.1%), and stomach (8.9%) are the most prevalent sites for the occurrence of GEP-NENs (Frilling et al., 2012). The aggressiveness of GEP-NENs depends on their primary site. Although small intestinal NENs are usually metastatic at the time of diagnosis, they show slow progression; in contrast, gastric and rectal NENs usually exhibit a low metastatic tendency but can advance rapidly once metastasis occurs. The majority of NENs are nonfunctional tumors that progress slowly and are frequently undetected due to a lack of specific symptoms or clinical signs, resulting in a delayed diagnosis. While the clinical presentation of carcinoid syndrome (CaS) associated with NENs is very heterogeneous, ranging from mild, often misdiagnosed symptoms, such as mild diarrhea and flushing, to severe manifestations including refractory diarrhea and fibrotic complications (Fröjd et al., 2007). A noteworthy finding is the correlation between the presence of CaS and the decreased overall survival (Fröjd et al., 2007; Fanciulli et al., 2020). Based on GEP-NENs’ heterogeneity, the WHO classifies GEP-NENs into three major categories: well-differentiated neuroendocrine tumors (NETs), poorly differentiated neuroendocrine carcinomas (NECs), and mixed neuroendocrine-non-neuroendocrine neoplasms (Rindi et al., 2022). GEP-NENs are the second most prevalent form of digestive cancer, with an increasing morbidity rate observed in recent decades (Yao et al., 2008). According to the data from the SEER program, the incidence of NENs is gradually increasing, partially because of advancements in diagnostic techniques and health awareness (Dasari et al., 2017).

The management of GEP-NENs has witnessed significant advancements over the past few decades. Despite these advancements, there are still many challenges to be faced. (1) Heterogeneity of NENs: the significant heterogeneity of GEP-NENs complicates the standardization of treatment protocols, thereby requiring personalized treatment approaches (Modica et al., 2022a,b). (2) Drug resistance: even the most advanced treatments may become ineffective over time, thus posing a significant clinical challenge of therapeutic resistance (Modica et al., 2022a,b). (3) Toxicity management: minimizing the toxicity of treatments while maintaining their efficacy is crucial; patients often experience adverse effects that can limit the use of certain therapies (Modica et al., 2022a,b). (4) Optimal sequencing and combination of therapies: identifying the most effective sequences and combinations of available treatments remains a challenge; clinical trials are needed to establish the best therapeutic algorithms (Modica et al., 2022a,b). (5) Predictive biomarkers: there is a lack of reliable biomarkers for predicting treatment response or monitoring disease progression (Modica et al., 2022a,b). Consequently, it is crucial to overcome the obstacles of tumor heterogeneity, improve early tumor detection, combat therapeutic resistance of tumors, identify optimal sequencing and combination of therapies, and identify predictive biomarkers for the advancement of this field.

The gut microbiota comprises various bacteria, fungi, archaea, and viruses that inhabit the intestinal tract, predominantly the colorectum (Lynch and Pedersen, 2016). The intestinal flora performs multiple functions as follows: it modulates the host’s metabolism, maintains the integrity of the intestinal barrier, participates in the metabolism of xenobiotics and drugs, and protects against gastrointestinal pathogens by regulating the host’s immune system; hence, it is crucial to maintain the balance of the intestinal flora (Cani, 2018; Schmidt et al., 2018; Vivarelli et al., 2019). The correlation between dysbiosis, or the imbalance of the intestinal flora, with various diseases, including cancer, has been widely investigated, and dysbiosis has been increasingly recognized as a significant factor in disease development (El Tekle and Garrett, 2023; Wong and Yu, 2023). Despite these findings, there has been limited research on the effect of the gut microbiome on GEP-NENs, primarily due to the lack of patients and the focus of most studies on gastrointestinal adenocarcinomas. In light of these challenges and the difficulties associated with conducting randomized controlled trials due to ethical and financial constraints, MR analysis offers a viable alternative. The MR analysis uses genetic variations as instrumental variables (IVs) to model interventions, thereby strengthening our capacity to draw more reliable conclusions regarding the effect of the intestinal microflora on the development of GEP-NENs (Porcu et al., 2019; Saunders et al., 2022).

In the present study, we conducted a bidirectional MR analysis to preliminarily investigate the potential causal impact of the intestinal flora on four common types of GEP-NENs, including pancreatic NENs, colorectal NENs, small intestinal NENs, and gastric NENs. The findings of this study could offer new perspectives regarding how the gut microbiota influences GEP-NEN development, which could enable to potentially identify a new target for screening and prevention strategies for these tumors.

## 2 Methods

### 2.1 Study design

**Figure 1:**
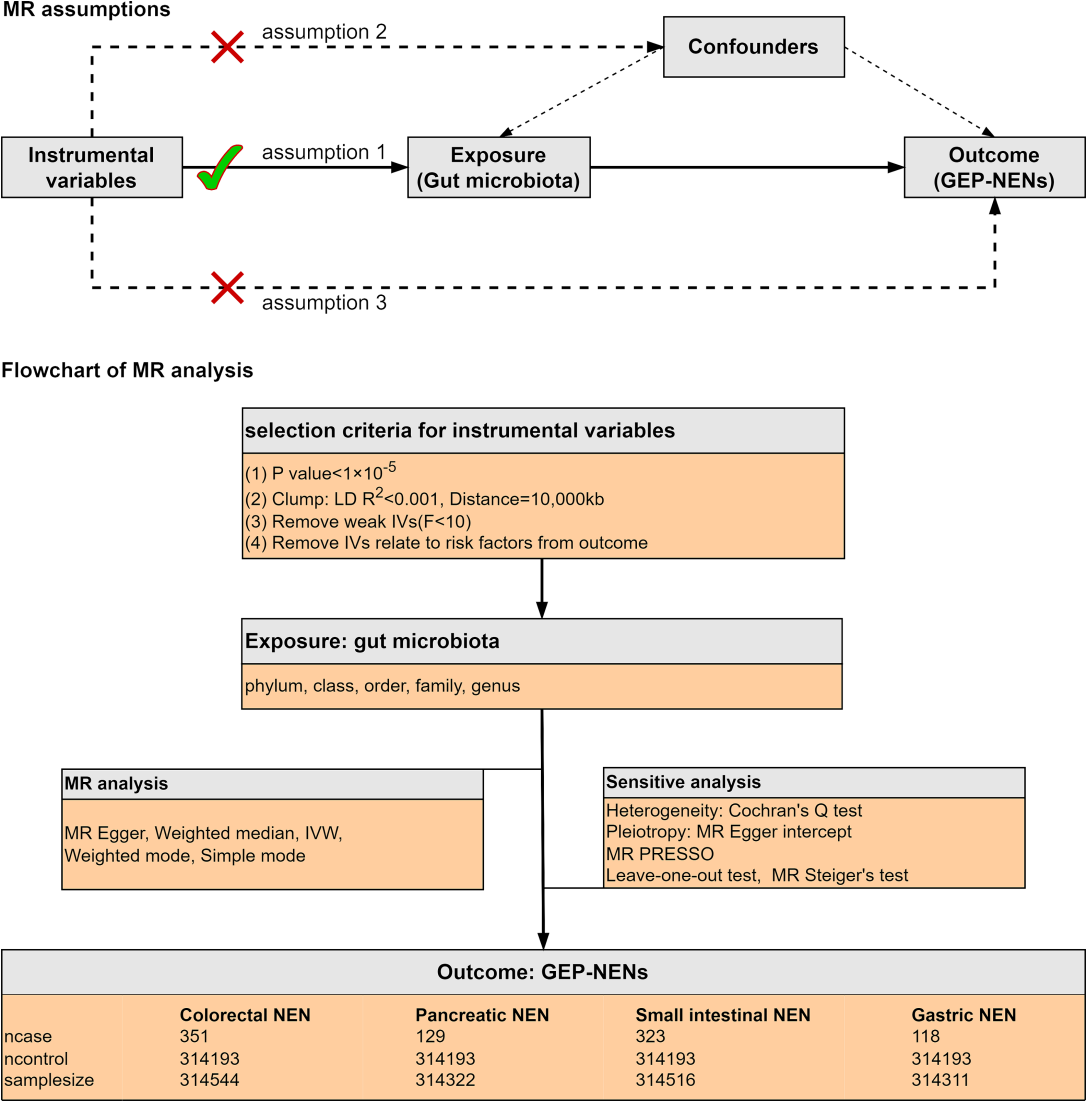
The current MR analysis was structured around three core assumptions, as shown in the figure. We conducted a two-sample MR study to assess the causal links between the intestinal microflora and four common subtypes of GEP-NENs. The inverse-variance weighted (IVW) method was used as the principal method for MR analysis. A range of sensitivity analyses were conducted to ensure the reliability of our results.

As shown in Figure 1, we conducted a two-sample MR analysis to assess the causal links between the intestinal flora and four prevalent GEP-NENs. This analysis adhered rigorously to the following fundamental principles of MR to confirm that our causal conclusions are valid. (1) IV relevance: we confirmed the correlation between the selected genetic variants and the intestinal flora, which ensured that these variants can serve as effective IVs for the exposure of interest (Sekula et al., 2016); (2) Independence from confounders: we confirmed that the selected genetic variants were not associated with any known confounders in the correlation between the intestinal flora and GEP-NENs, which enabled to maintain the integrity of our causal inference (Jia et al., 2023); and (3) Exclusivity of the exposure pathway: we ascertained that the influence of the selected genetic variants on the occurrence of GEP-NENs was mediated exclusively through the gut microbiota, with no direct effects or through alternate biological pathways (Jin et al., 2023).

### 2.2 Data sources

The summary statistics for the intestinal flora were derived from the largest genome-wide association study (GWAS) performed by the MiBioGen consortium,^1^ which comprised 18,340 participants across 24 cohorts. The majority of the participants (14,306 individuals) were of European descent (Kurilshikov et al., 2021). Through coordinated efforts in 16S rRNA gene sequencing and genetic typing, the GWAS identified 211 bacterial taxa, which included 9 phyla, 16 classes, 20 orders, 35 families, and 131 genera. After excluding 15 taxa (3 families and 12 genera) associated with unidentified groups, 196 bacterial taxa were included in the MR study. Detailed information regarding the microbiota dataset is available in the original article (Kurilshikov et al., 2021).

Summary statistics for GEP-NENs were sourced from the FinnGen research project,^2^ which is a comprehensive initiative based on the Finnish National Biobank Network. Participant recruitment for the FinnGen project spanned from 2017 through 2023, and the project has a dataset of 315,114 participants. Within this cohort, the study identified 129 individuals diagnosed with pancreatic NENs, 351 with colorectal NENs, 323 with small intestinal NENs, and 118 with gastric NENs. These four GEP-NEN types had a common control group that comprised the entire participant pool of 315,114 individuals. Table 1 shows the details of these datasets.

**Table 1:** Details regarding the data source of the gut microbiota and four common GEP-NENs.

Trait	Consortium	Year	Case	Control	Samplesize	Population
Gut microbiota	MiBioGen	2021	18,340	–	18,340	European
Pancreatic NEN	FinnGen (R10)	2023	129	314,193	314,322	European
Colorectal NEN	FinnGen (R10)	2023	351	314,193	314,544	European
Small intestinal NEN	FinnGen (R10)	2023	323	314,193	314,516	European
Gastric NEN	FinnGen (R10)	2023	118	314,193	314,311	European

### 2.3 IV selection

To identify suitable genetic IVs for our analysis, we used a range of selection criteria to ensure robustness and relevance of the obtained results. (1) Threshold for inclusion: because of the scarcity of genetic variants that can meet the genome-wide significance threshold (*p* < 5 × 10^−8^), we chose to apply a more lenient threshold (*p* < 1 × 10^−5^). This adjustment, based on insights from prior studies (Kurilshikov et al., 2021; Li et al., 2022, 2023; Zeng et al., 2023), aimed to encompass a broader set of variants likely enriched for associations, thereby enabling a more exhaustive exploration of potential links (Sanna et al., 2019). (2) Clumping for independence: by utilizing the reference panel from the 1,000 Genome Project focused on European samples, we conducted a clumping process with a threshold of R^2^ < 0.001 and a window size of 10,000 kb to remove variants in strong linkage disequilibrium (LD). This step ensured that the selected single nucleotide polymorphisms (SNPs) are independent, thereby mitigating the risk of overestimating associations. (3) Exclusion of ambiguous and palindromic SNPs: variants with non-concordant alleles or palindromic SNPs, which could introduce bias or ambiguity, were excluded from consideration in this analysis. (4) Instrument strength assessment: the strength of each SNP was assessed using the F-statistic, calculated using the formula F = (*n* – k – 1)^2^/(1 – R^2^), where “n” denotes the sample size of the exposure dataset, “k” represents the number of SNPs, and “R^2^” is the proportion of variance in the exposure that is explained by the genetic factors. SNPs with an *F*-value less than 10 were deemed weak IVs and subsequently excluded from the analysis (Burgess et al., 2017). The proportion of variance explained (R^2^) was determined using the formula: R^2^ = 2 × eaf × (1 – eaf) × beta^2^/(2 × eaf × (1 – eaf) × beta^2^ + 2 × eaf × (1 – eaf) × *n* × se^2^) (Pierce et al., 2011). This meticulous approach to select IVs was designed to maximize the validity and interpretability of our MR findings, and it provided a robust basis for evaluating the causal effect of the intestinal flora on GEP-NENs.

### 2.4 MR analysis

To assess the causal relationship between the intestinal flora and GEP-NENs, we used five different MR methods: MR-Egger, weighted median (WM), IVW, weighted mode, and simple mode. The IVW method, which was the primary method of analysis, assumes that all selected SNPs function as valid IVs (Burgess et al., 2013). This method calculates the effect ratio of each IV (genetic variant) on the exposure and outcome. By weighting these ratios based on their variance and then averaging them, this method estimates the overall causal effect of the exposure on the disease outcome. The main advantage of the IVW method is that it combines information from multiple IVs, which enhances the precision and robustness of the effect estimate. However, a limitation of this method is that the presence of invalid IVs or pleiotropy can introduce bias into the results. The IVW method can be applied using either fixed-effects or multiplicative random-effects models, depending on whether the SNP effects are consistent. Unlike the IVW, the MR-Egger method is used to detect and adjust for horizontal pleiotropy by introducing an intercept in the regression model; this enables to identify whether genetic variants influence the outcome through pathways other than the exposure. The advantages of this method include the ability to provide more reliable causal estimates by accounting for pleiotropy, thereby making the analysis more robust. However, the disadvantages of this method are lower statistical power, requirement for larger sample sizes and more IVs, stricter assumptions, and potential inaccuracy for weak instruments (Burgess and Thompson, 2017). We also conducted validation using the Benjamini-Hochberg correction to control the FDR. The results with a corrected *p*-value less than 0.05 were regarded as indicative of a significant causal effect. However, it is important to note that microbiota associations with a *p*-value less than 0.05, although not significant after adjustment, may still suggest a potential causal link with GEP-NENs.

### 2.5 Sensitivity analysis

To assess the stability and reliability of our conclusions regarding the causal links between the intestinal flora and GEP-NENs, we performed a range of sensitivity analyses as follows. (1) Heterogeneity evaluation: Cochran’s Q test was used to determine the heterogeneity among the IVs used in the analysis. A *p*-value less than 0.05 indicated significant heterogeneity, prompting the application of the random-effects IVW method to accommodate this variability. (2) Detection of horizontal pleiotropy: the MR-Egger intercept and MR-PRESSO tests were used to detect potential horizontal pleiotropy, where IVs might affect the outcome through pathways unrelated to the exposure of interest. MR-PRESSO, a robust method in MR analysis, is designed to detect and correct for horizontal pleiotropy by identifying and removing outlier IVs. It enhances the reliability of causal effect estimates but involves a complex and computationally intensive process, and its effectiveness depends on the initial selection of IVs. (3) Directionality confirmation: the MR-Steiger model was used as an additional check for the overall directionality of the estimated causal association, which ensured the consistency of the analysis findings. A “TRUE”’ outcome from the test signifies that the direction of the association aligns with the hypothesized causal pathway. (4) Influential SNP assessment: we conducted a leave-one-out sensitivity test to evaluate the effect of isolated SNPs on the comprehensive analysis. This approach involves sequentially excluding each SNP from the analysis to observe its impact on the overall findings, helping to identify any SNPs that disproportionately affect the causal estimate. These sensitivity analyses were critical to confirm the integrity of our MR study and ensured that the observed correlations are not artifacts of statistical heterogeneity, pleiotropy, or outlier SNPs. By rigorously testing these aspects, we aimed to obtain solid evidence that supports the causal effect of the intestinal microflora on GEP-NENs.

### 2.6 Reverse MR analysis

To assess reverse causality between the intestinal flora and GEP-NENs, a reverse MR analysis was performed by considering GEP-NENs as the exposure and the gut microbiota as the outcome. A two-sample MR study was performed using the same approaches and adhering to the three hypotheses described earlier in the study.

### 2.7 Statistical analysis

All statistical analyses were performed using RStudio version 4.3.2, with the TwoSampleMR package version 0.5.10.

## 3 Results

### 3.1 A summary of IVs in the gut microbiota

By using a lenient threshold screening (*p* < 1 × 10^−5^), along with harmonization and validation of F-statistics, we identified multiple SNPs as IVs across 196 bacterial taxa. All the retained SNPs had an F-statistic greater than 10, as shown in Supplementary Table S1; this finding demonstrated a strong link between the IVs and the related bacterial taxa and confirmed the absence of weak instrumental bias in our research. Additionally, after excluding SNPs identified as pleiotropic through the MR-PRESSO outlier test and the MR-Egger intercept test, our analysis confirmed that the IVs did not show signs of horizontal pleiotropy, with nonsignificant *p*-values (*p* > 0.05) in both the MR-Egger intercept and MR-PRESSO tests, as detailed in Supplementary Table S3. This rigorous validation process underscores the reliability of our IVs in examining the genetic determinants of microbiota composition without interference from unrelated genetic pathways.

### 3.2 Causal effect of the intestinal flora on the four types of GEP-NENs

**Figure 2:**
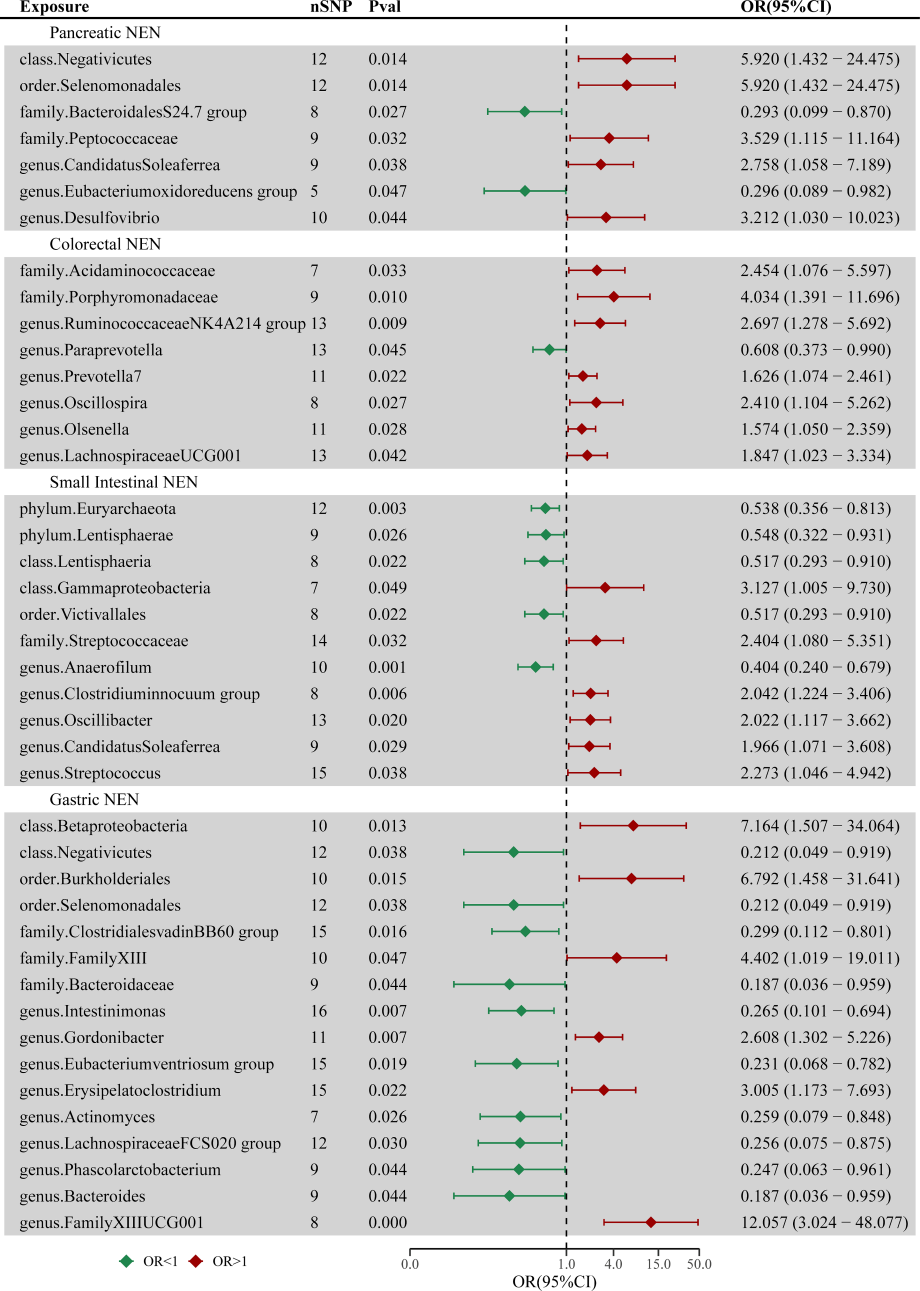
Forest plot of casual associations between the intestinal flora and four types of GEP-NENs by using the IVW method. OR, odds ratio; CI, confidence interval.

The findings of the MR analysis for the casual association between the intestinal flora and the four common types of GEP-NENs are shown in Figure 2 and Supplementary Table S2.

#### 3.2.1 Pancreatic NENs

The MR analysis revealed 7 taxa associated with pancreatic NENs, as shown in Supplementary Figure S1. Genetically inferred risk for pancreatic NENs was associated with Negativicutes (*p* = 0.014), Selenomonadales (*p* = 0.014), Peptococcaceae (*p* = 0.032), Candidatus Soleaferrea (*p* = 0.038), and Desulfovibrio (*p* = 0.044). Conversely, taxa such as the Bacteroidales S24-7 group (*p* = 0.027) and the *Eubacterium oxidoreducens* group (*p* = 0.047) exhibited protective roles against pancreatic NENs.

#### 3.2.2 Colorectal NENs

Eight microbial taxa were identified with causal associations to colorectal NENs, as shown in Supplementary Figure S2. Microbial communities such as Acidaminococcaceae (*p* = 0.033), Porphyromonadaceae (*p* = 0.010), Ruminococcaceae NK4A214 group (*p* = 0.009), Prevotella 7 (*p* = 0.022), Oscillospira (*p* = 0.027), Olsenella (*p* = 0.028), and Lachnospiraceae UCG-001 (*p* = 0.042) were found to potentially increase the risk for the occurrence of colorectal NENs, whereas Paraprevotella (*p* = 0.045) might reduce this risk.

#### 3.2.3 Small intestinal NENs

Eleven taxa exhibited causal relationships between the gut microbiota and small intestinal NENs across the two datasets, as shown in Supplementary Figure S3. Five taxa, including Euryarchaeota (*p* = 0.003), Lentisphaerae (*p* = 0.026), Lentisphaeria (*p* = 0.022), Victivallales (*p* = 0.022), and Anaerofilum (*p* = 0.006), were inversely correlated with the risk of GEP-NEN development. In contrast, Gammaproteobacteria (*p* = 0.049), Streptococcaceae (*p* = 0.032), *Clostridium innocuum* group (*p* = 0.006), Oscillibacter (*p* = 0.020), Candidatus Soleaferrea (*p* = 0.030), and Streptococcus (*p* = 0.038) were directly associated with the risk of GEP-NEN occurrence. Euryarchaeota remained a significant taxon even after adjusting the *p*-value (p_adj = 0.029).

#### 3.2.4 Gastric NENs

Sixteen bacterial taxa were associated with the occurrence of gastric NENs, as shown in Supplementary Figure S4. The IVW method indicated a causal link of Betaproteobacteria (*p* = 0.013), Burkholderiales (*p* = 0.015), Family XIII (*p* = 0.047), Gordonibacter (*p* = 0.007), Erysipelatoclostridium (*p* = 0.022), and Family XIII UCG-001 (*p* = 0.0004) with the risk of gastric NEN occurrence. Additionally, Negativicutes (*p* = 0.038), Selenomonadales (*p* = 0.038), Clostridiales vadin BB60 group (*p* = 0.016), Bacteroidaceae (*p* = 0.044), Intestinimonas (*p* = 0.007), *Eubacterium ventriosum* group (*p* = 0.019), Actinomyces (*p* = 0.026), Lachnospiraceae FCS020 group (*p* = 0.030), Phascolarctobacterium (*p* = 0.044), and Bacteroides (*p* = 0.044) were the protective factors against gastric NENs. Family XIII UCG-001 remained significant even after adjusting the *p*-value (p_adj = 0.0498).

### 3.3 Sensitivity analysis

Sensitivity analyses were conducted to confirm the integrity and stability of our findings. The outcomes of Cochran’s Q test indicated consistency among the IVs, thus suggesting homogeneity. Moreover, except for the genus Prevotella 9, the MR-Egger intercept test did not show any indication of pleiotropic effects (all *p*-values >0.05), which was corroborated by the results of the MR-PRESSO test (all *p*-values >0.05). These analyses proved that the IVs are likely to exert their impact on the risk of GEP-NENs only through the gut microbiota, without any evidence of significant deviation through alternate pathways. Supplementary Table S3 shows the detailed results of these analyses. Supplementary Figures S5–S8 present a comprehensive synopsis of the causal associations exerted by specific taxa on the four types of GEP-NENs, as determined by the leave-one-out analysis. This methodical approach reaffirmed the stability of our results, excluding the possibility that any single taxon disproportionately influenced the overall causal inference. The MR-Steiger test further validated the causal effect of the intestinal flora on GEP-NENs, confirming that the impact of the intestinal flora aligns with the expected causal direction. Thus, these sensitivity analyses reinforced the robustness of our conclusion that the gut microbiota has a causal relationship with the occurrence of GEP-NENs.

### 3.4 Causal effect of GEP-NENs on the intestinal flora

**Figure 3:**
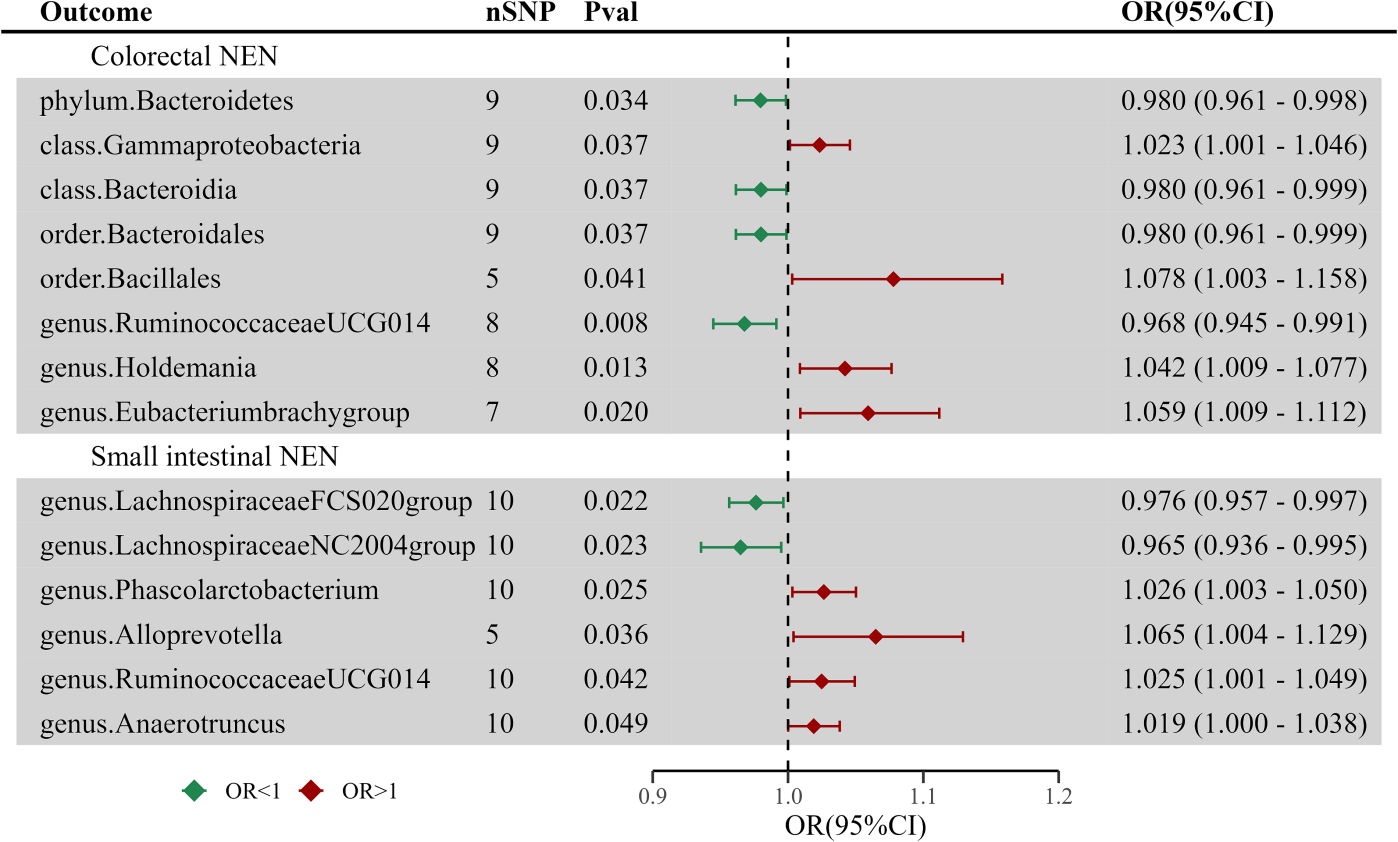
Forest plot of casual links between GEP-NENs and the intestinal microbiota by the IVW method.

A reverse MR analysis was performed to assess the possibility that different types of GEP-NENs might have a causal effect on the composition of the intestinal microflora. This reverse MR analysis followed the same procedures as the initial MR analysis. The results showed that colorectal NENs and small intestinal NENs were associated with variations in 8 and 6 microbial taxa of the intestinal flora, respectively. This finding suggests a potential bidirectional link between these NENs and the specific groups of gut microorganisms, as shown in Supplementary Tables S4–S6 and Figure 3. After adjusting for FDR correction, no significant causal links were observed between GEP-NENs and the intestinal flora. For pancreatic NENs and gastric NENs, even though we utilized a more lenient threshold (*p* < 1 × 10^−5^), there were very few effective SNPs that could not complete sensitivity analysis; thus, we excluded the results for these two types of GEP-NENs.

## 4 Discussion

The intricate interplay between the gut microbiota and human health has become a central theme in modern medical research, with extensive studies on elucidating its critical role in the development of various illnesses, including cancer (Vimal et al., 2020; Fan et al., 2021). Dysbiosis, a state of microbial imbalance within the gut, is involved in the etiology of different types of tumors. This disruption in microbial harmony is thought to influence cancer development through mechanisms such as chronic inflammation, alteration of the gut barrier, and modulation of host immune responses. Although substantial research efforts have been directed toward exploring the correlations between the intestinal flora and gastrointestinal adenocarcinomas, the association of the gut microbiota with GEP-NENs remains relatively underexplored. Although *Helicobacter pylori* appears to be associated with the development of gastric NENs, there is currently insufficient data regarding the role of the gut microbiota in the development of other GEP-NENs (Vitale et al., 2021). Preliminary findings indicate a decrease in the abundance of *Faecalibacterium prausnitzii* in patients with midgut NENs and inflammatory bowel disease (Vitale et al., 2021). Moreover, this association between gut flora and GEP-NENs could be contextualized even in the gender difference that exist in NENs (Muscogiuri et al., 2020; Liccardi et al., 2024). However, the causal relationship between these events has not been definitely established. Thus, there is an imminent need to clarify the role of the gut microbiota in the development of GEP-NENs. In the present research, we performed a MR analysis to determine the potential causal effect of the intestinal flora on GEP-NENs by leveraging the largest available GWAS datasets on both intestinal flora and GEP-NENs. The results provide strong genetic evidence indicating that alterations in the composition of specific gut microorganisms significantly affect the risk and progression of GEP-NENs. This finding indicates a significant genetic relationship between the composition of gut microorganisms and the development of these tumors.

The investigation of the correlation between the intestinal flora and GEP-NENs is a burgeoning field and has yielded intriguing findings. Hu et al. assayed fecal samples from patients with rectal NENs and found disruption of the intestinal microflora and metabolic features in these patients (Hu et al., 2022). Mohamed et al. discovered significant variations in the composition of bacterial and fungal species between patients with GEP-NENs and healthy individuals; their study highlighted an increase in fungi, particularly Candida, Ascomycota, and Saccharomycetes species (Mohamed et al., 2022). Massironi et al. observed bacterial colonization in both intestinal NEN and pancreatic NEN tissues, with a marked increase in bacterial infiltration in pancreatic tumors as compared to that in nontumoral pancreatic tissues (Pushalkar et al., 2018; Thomas et al., 2018; Massironi et al., 2022). This finding suggests not only the presence of a pancreatic microbiota in healthy and diseased states but also its potential involvement in tumor pathology. Mulders et al. also reported a reduced diversity and richness in the gut microbiome of midgut NEN patients as compared to that of controls, reinforcing the concept of a significant association between alterations in the gut microbiota and the occurrence of GEP-NENs (Mulders et al., 2024).

Our study on the complex microbiome of the gastrointestinal tract revealed a significant and protective association between Euryarchaeota and small intestinal NENs, shedding light on the nuanced interplay between microbial inhabitants and their host. Euryarchaeota, a phylum of archaea, is the cornerstone of the gut’s microbial ecosystem, and the members of this phylum participate in key metabolic processes essential for maintaining host health (Bäckhed et al., 2005; Koskinen et al., 2017). This group shows remarkable diversity and include methanogens known for methane production, a common phenomenon within the intestinal milieu. Methanogenic archaea play a pivotal role by engaging in synergistic interactions with bacteria to facilitate the production of short-chain fatty acids (SCFAs) (Lurie-Weinberger and Gophna, 2015). These compounds, particularly propionate and butyrate, are known for their anticancer effects in the gastrointestinal tract (Louis et al., 2014). SCFAs have been proven to regulate lipid metabolism (He et al., 2020), and further studies have revealed that modulation of lipid metabolism would probably become a new method in NEN therapy (Modica et al., 2022a,b, 2024a,b). These findings offer a potential mechanistic explanation for the protective effects observed in our study. The intricate associations between Euryarchaeota and bacterial communities underscore the delicate balance of the gut microbiome, and disturbances in this equilibrium—known as dysbiosis—are linked with an elevated risk of cancers such as breast and colorectal cancer (Mikó et al., 2019; Coker et al., 2020). Our findings reflect these observations, highlighting a positive correlation between methanogenic archaea and bacterial populations in the human body (Hansen et al., 2011), a mutual symbiosis further validated in both human studies and humanized gnotobiotic mouse models (Samuel and Gordon, 2006). The presence of methanogens correlates with increased gastrointestinal levels of beneficial SCFAs (Goodrich et al., 2014; Kumpitsch et al., 2021), suggesting that the interaction of Euryarchaeota with bacteria not only favors a harmonious microbial environment but also potentially contributes to tumor suppression. Although our research provides foundational perceptions into the protective correlations between these ancient microorganisms and GEP-NENs, the precise mechanisms remain unelucidated. Further studies are required to confirm the potential role of Euryarchaeota in the development and progression of small intestinal NENs. These findings could have significant clinical implications for the prevention and treatment of GEP-NENs.

The genus Family XIII UCG-001, a relatively obscure group in the Firmicutes phylum, has attracted interest because of its unique association with human health. Prior research has intriguingly linked Family XIII UCG-001 with facial skin aging. Additionally, a recent MR analysis revealed that Family XIII UCG-001 is a risk factor for heart failure, suggesting its potential impact on systemic biological processes beyond the gut microbiome (Chen et al., 2024; Pang et al., 2024). Despite these interesting observations, the impact of Family XIII UCG-001 on gastrointestinal diseases, particularly gastric NENs, remains largely unknown. The significance of the intestinal flora in the development and progression of gastric NENs has been increasingly recognized, prompting a need to gain more detailed understanding of the contributions of specific microbial taxa. Our present study is a pioneering investigation on the role of Family XIII UCG-001 in gastric NENs and showed a potentially hazardous causal relationship. This discovery not only enriches our understanding of how the microbiome affects gastric NENs but also underscores the necessity for further studies on the underlying mechanisms of this association. The identification of a harmful link between Family XIII UCG-001 and gastric NENs could have significant implications for developing novel diagnostic markers and therapeutic strategies and can potentially guide interventions focused on adjusting the intestinal flora to reduce the risk or slow the progression of gastric NENs.

Although our research did not conclusively show a causal link between the intestinal flora and the occurrence of pancreatic or colorectal NENs, this absence of direct causality should not be misconstrued as negating the significant role of the intestinal flora in tumor development and progression. Several observational research studies have revealed significant differences in the composition of the intestinal microflora between patients with pancreatic and colorectal NENs and healthy controls, suggesting a link between microbiota dysbiosis and these tumor types (Hu et al., 2022; Massironi et al., 2022; Chen et al., 2024). The intestinal microflora may serve as a crucial intermediary in the cascade of events leading to tumor formation and growth. MR studies are specifically designed to deduce the potential causal effects of exposures on outcomes by using genetic variants as IVs; thus, the observed microbiota dysbiosis in pancreatic and colorectal NEN patients may reflect the underlying pathological processes rather than serve as a direct causative factor. A hypothesis for the absence of a direct causal link is that this microbiome could be part of a scale-free network, contributing collectively to the pathogenesis of GEP-NENs. Individually, each microbial taxon might have a minimal effect, but together, they could exert significant effects, similar to the concept of polygenic risk scores in the study of complex traits.

To the best of our knowledge, this is the first MR study focused on the correlation between the gut microbiota and the occurrence of GEP-NENs. Our study could serve as a valuable reference to identify biomarkers and potential targets for intervention within this disease cluster, thereby enabling to address clinical challenges. Additionally, our findings may offer new insights and directions for understanding the underlying mechanisms of GEP-NEN development. Nevertheless, the present study has some limitations. The granularity of the data, confined to the genus level, restricts our ability to investigate the causal relationships at the more specific species level, potentially overlooking finer microbial influences on GEP-NENs. Moreover, the sensitivity analyses and detection of horizontal pleiotropy in our research were restricted by a lack of genetic variants used as IVs, with the SNPs not attaining the traditional GWAS significance threshold (*p* < 5 × 10^−8^). To reduce the risk of false positives, we applied FDR correction. Additionally, although the GWAS meta-analysis for the intestinal flora primarily included participants of European descent, the possibility of population stratification remains a concern. This factor could influence the generalizability of our results to other populations. Future MR studies analyzing the causal effect of the intestinal microflora on GEP-NENs should consider the inclusion of a more diverse array of populations, spanning both European and non-European backgrounds, to strengthen the generalizability of the results. Expanding the scope of research through this approach will be crucial to advance our understanding of GEP-NENs and could enable to develop universally effective interventions.

## 5 Conclusion

Our present study reveals significant associations that emphasize the potential role of the gut microbiota in the development of GEP-NENs. These findings may contribute to recognize valuable biomarkers for the early, noninvasive detection of GEP-NENs. Moreover, our results could enable to identify viable targets for therapeutic interventions in treating this disease, offering new avenues for both the diagnosis and treatment of GEP-NENs.

## Data availability statement

The data presented in the study are deposited in the Google Drive repository, accession link https://drive.google.com/file/d/1HnzVg3cLzRYQMLb-dGct5Fv05SfwgA8z/view?usp=drive_link.

## Author contributions

C-yZ: Writing – original draft, Writing – review & editing. S-jJ: Writing – original draft, Writing – review & editing. J-jC: Writing – original draft, Writing – review & editing. YX: Writing – original draft, Writing – review & editing. X-yW: Writing – original draft, Writing – review & editing. RL: Writing – original draft, Writing – review & editing. Z-wM: Conceptualization, Data curation, Formal analysis, Funding acquisition, Investigation, Methodology, Project administration, Resources, Software, Supervision, Validation, Visualization, Writing – original draft, Writing – review & editing.

## Footnotes

^1^ www.mibiogen.org

^2^ https://r10.finngen.fi/
