# Supplementary material for: Investigating the causal relationship between gut microbiota and gastroenteropancreatic neuroendocrine neoplasms: a bidirectional Mendelian randomization study
Source: Front Microbiol. 2024 Aug 13;15:1420167. doi: 10.3389/fmicb.2024.1420167 (PMC11347282; doi:10.3389/fmicb.2024.1420167)
Supplement: Supplementary file 2 [file Table_2.DOCX]

**STROBE-MR checklist of recommended items to address in reports of Mendelian randomization studies**^1^ ^2^

| **Item No.** | **Section** | **Checklist item** | **Page No.** | **Relevant text from manuscript** |
| --- | --- | --- | --- | --- |
| 1 | **TITLE and ABSTRACT** | Indicate Mendelian randomization (MR) as the study’s design in the title and/or the abstract if that is a main purpose of the study | 1 | Title: Causal links between gut microbiota and gastroenteropancreatic neuroendocrine neoplasms: a bidirectional mendelian randomization study  Abstract:  Methods: A two-sample bidirectional Mendelian randomization study was conducted using the largest existing gut microbiota and four types of GEP-NENs genome-wide association studies (GWAS). |
|  | **INTRODUCTION** |  |  |  |
| 2 | **Background** | Explain the scientific background and rationale for the reported study. What is the exposure? Is a potential causal relationship between exposure and outcome plausible? Justify why MR is a helpful method to address the study question | 2,3 | Dysbiosis of gut microbiota has been widely studied in various types of digestive disease, including cancer. Gut microbiota dysbiosis has been recognized as a hallmark of cancer by the scientific community (El Tekle and Garrett, 2023; Wong and Yu, 2023). However, to date, partly due to the relatively low number of patients, most of the studies have focused on the association between gut microbiota and gastrointestinal adenocarcinomas, very little is known about GEP-NENs. The causal relationship and mechanisms between gut microbiota and GEP-NENs are still unclear, which poses obstacles to the prevention and treatment of GEP-NENs. Thus, it is imperative to study the causal link between gut microbiota and GEP-NENs. MR analysis employs genetic variation as instrumental variables(IVs) to model interventions, enhancing our ability to make more confident inferences regarding the influence of a factor on disease occurrence (Porcu et al., 2019; Saunders et al., 2022). |
| 3 | **Objectives** | State specific objectives clearly, including pre-specified causal hypotheses (if any). State that MR is a method that, under specific assumptions, intends to estimate causal effects | 3 | This study aims to investigate the causal relationship between gut microbiota and GEP-NENs using bidirectional Mendelian randomization analysis. |
|  | **METHODS** |  |  |  |
| 4 | **Study design and data sources** | Present key elements of the study design early in the article. Consider including a table listing sources of data for all phases of the study. For each data source contributing to the analysis, describe the following: | 5,6 | Table listing sources of data for all phases of the study: Table 1 |
|  | a) | Setting: Describe the study design and the underlying population, if possible. Describe the setting, locations, and relevant dates, including periods of recruitment, exposure, follow-up, and data collection, when available. | 5,6 | GWAS summary data for gut microbiota was accessed from the MiBioGen consortium, comprising 18,340 participants from 24 cohorts. The majority of participants in the study were of European descent (N=14306) (Kurilshikov et al., 2021).  GWAS summary data for GEP-NENs was accessed from the FinnGen research project, a comprehensive initiative drawing upon the Finnish National Biobank Network. Recruitment for FinnGen spanned from 2017 through 2023, assembling a dataset of 315,114 participants. |
|  | b) | Participants: Give the eligibility criteria, and the sources and methods of selection of participants. Report the sample size, and whether any power or sample size calculations were carried out prior to the main analysis | 5,6 | GWAS summary data for gut microbiota was accessed from the MiBioGen consortium, with a sample size of 18,340.  GWAS summary data for four types of GEP-NENs was accessed from the FinnGen research project, within this cohort, the study identified 129 individuals diagnosed with pancreatic NENs, 351 with colorectal NENs, 323 with small intestinal NENs, and 118 with gastric NENs. Notably, these four types of GEP-NENs utilized a common control group, encompassing the entire participant pool of 315,114 individuals.  Due to the use of existing data, we did not perform power calculations. |
|  | c) | Describe measurement, quality control and selection of genetic variants | 5,6 | To identify suitable genetic IVs for our analysis, we applied a series of selection criteria to ensure robustness and relevance.(1) Threshold for Inclusion( p < 1 × 10^−5^ ), (2) Clumping for Independence(R^2^ < 0.001, window size = 10,000 kb), (3) Exclusion of ambiguous and palindromic SNPs, (4) Instrument Strength Assessment(F>10) |
|  | d) | For each exposure, outcome, and other relevant variables, describe methods of assessment and diagnostic criteria for diseases | 5,6 | By coordinated 16S rRNA gene sequencing profiles and genetic typing data, a total of 211 taxa were identified in the GWAS. Encompassing 9 phyla, 16 classes, 20 orders, 35 families, and 131 genera. After the exclusion of 15 taxa associated with unidentified groups (comprising 12 genera and 3 families), 196 bacterial taxa were incorporated into the MR evaluation. Comprehensive information regarding the microbiota dataset can be found in the original investigation (Kurilshikov et al., 2021).  The four types of GEP-NENs refer to the definitions of the 10th edition of the International Classification of Diseases (ICD-10). |
|  | e) | Provide details of ethics committee approval and participant informed consent, if relevant | 5,6 | The GWAS data used in this study were all from publicly available databases. The summary statistics of gut microbiota abundance and GEP-NENs do not contain any personal information, and each GWAS has received ethical approval from the relevant ethical review board. |
| 5 | **Assumptions** | Explicitly state the three core IV assumptions for the main analysis (relevance, independence and exclusion restriction) as well assumptions for any additional or sensitivity analysis | 3,4 | The Mendelian randomization design consisted of three components. (1) Instrumental variable relevance: We confirmed a strong association between the selected genetic variants and the gut microbiota, ensuring these variants can serve as effective IVs for our exposure of interest (Sekula et al., 2016); (2) Independence from confounders: We verified that the chosen genetic variants were not associated with any known confounders of the relationship between gut microbiota and GEP-NENs, maintaining the integrity of our causal analysis (Jia et al., 2023); (3) Exclusivity of the exposure pathway: We ascertained that the influence of genetic variants on GEP-NENs occurrence was mediated exclusively through the gut microbiota, with no direct effects or via alternate biological pathways (Jin et al., 2023). Figure 1 outlines the design and flowchart of the Mendelian randomization study. |
| 6 | **Statistical methods: main analysis** | Describe statistical methods and statistics used |  |  |
|  | a) | Describe how quantitative variables were handled in the analyses (i.e., scale, units, model) | 7 | To assess the causal link between gut microbiota and GEP-NENs, we implemented five MR methodologies: IVW, MR-Egger, weighted median (WM), weighted mode, and simple mode. The IVW method, serving as our primary analytic approach. |
|  | b) | Describe how genetic variants were handled in the analyses and, if applicable, how their weights were selected | 6,7 | To identify suitable genetic IVs for our analysis, we applied a series of selection criteria to ensure robustness and relevance.(1) Threshold for Inclusion( p < 1 × 10−5 ), (2) Clumping for Independence(R2 < 0.001, window size = 10,000 kb), (3) Exclusion of ambiguous and palindromic SNPs, (4) Instrument Strength Assessment(F>10) |
|  | c) | Describe the MR estimator (e.g. two-stage least squares, Wald ratio) and related statistics. Detail the included covariates and, in case of two-sample MR, whether the same covariate set was used for adjustment in the two samples | 7 | Two-Sample Mendelian randomization  We primarily used the inverse variance weighted (IVW) method, supplemented by MR-Egger, weighted median, weighted mode, and simple mode methods, to ensure comprehensive causal inference. |
|  | d) | Explain how missing data were addressed | 6 | The GWAS data from the finngen database contains a small amount of missing SNP data, which we excluded in our analysis. |
|  | e) | If applicable, indicate how multiple testing was addressed | 7 | We performed validation using the Benjamini-Hochberg correction which was used to control false discovery rate(FDR). |
| 7 | **Assessment of assumptions** | Describe any methods or prior knowledge used to assess the assumptions or justify their validity | 7 | The strength of each SNP was evaluated using the F-statistic, calculated as F = (n – k – 1)^2^ / (1 – R^2^), where 'n' represents the sample size of the exposure dataset, 'k' the number of SNPs, and 'R^2^' the proportion of variance in exposure explained by the genetic factors. SNPs yielding an F-value below 10 were deemed weak IVs and excluded from our analysis (Burgess et al., 2017). The proportion of variance explained (R^2^) was determined using the formula: R^2^ = 2 × EAF × (1 – EAF) × beta^2^ / (2 × EAF × (1 – EAF) × beta^2^ + 2 × EAF × (1 – EAF) × n × se^2^) (Pierce et al., 2011). |
| 8 | **Sensitivity analyses and additional analyses** | Describe any sensitivity analyses or additional analyses performed (e.g. comparison of effect estimates from different approaches, independent replication, bias analytic techniques, validation of instruments, simulations) | 7,8 | Sensitivity analysis, including Cochran’s Q test, MR Egger intercept test, MR Pleiotropy Residual Sum and Outlier (MR-PRESSO), and leave-one-out analysis, were conducted to ensure the robustness of our findings. |
| 9 | **Software and pre-registration** |  |  |  |
|  | a) | Name statistical software and package(s), including version and settings used | 8 | All statistical analyses were conducted using R version 4.3.2, using the TwoSampleMR version 0.5.10. |
|  | b) | State whether the study protocol and details were pre-registered (as well as when and where) |  | This study protocol and details were not pre-registered. |
|  | **RESULTS** |  |  |  |
| 10 | **Descriptive data** |  |  |  |
|  | a) | Report the numbers of individuals at each stage of included studies and reasons for exclusion. Consider use of a flow diagram |  | The GWAS data used in this study is derived from the GWAS data of existing studies, so it is not reported. |
|  | b) | Report summary statistics for phenotypic exposure(s), outcome(s), and other relevant variables (e.g. means, SDs, proportions) |  | We report summary statistics for gut microbiota and four types of GEP-NENs data in Table 1. |
|  | c) | If the data sources include meta-analyses of previous studies, provide the assessments of heterogeneity across these studies |  | The data sources didn’t include meta-analyses of previous studies. |
|  | d) | For two-sample MR:  i.  Provide justification of the similarity of the genetic variant-exposure associations between the exposure and outcome samples  ii.  Provide information on the number of individuals who overlap between the exposure and outcome studies |  | A genome-wide significance threshold screening (P< 1×10^–5^), harmonization, and verification of F statistics have identified multiple SNPs as IVs in 196 bacterial taxa. In all maintained SNPs, the F-statistic is greater than 10, indicating sufficient power of correlation between the IVs and the associated bacterial taxon ([Supplementary Table S1](https://www.ncbi.nlm.nih.gov/pmc/articles/PMC10702359/#TS2)). |
| 11 | **Main results** |  |  |  |
|  | a) | Report the associations between genetic variant and exposure, and between genetic variant and outcome, preferably on an interpretable scale |  | Table S1, Table S2 |
|  | b) | Report MR estimates of the relationship between exposure and outcome, and the measures of uncertainty from the MR analysis, on an interpretable scale, such as odds ratio or relative risk per SD difference |  | Table S2 |
|  | c) | If relevant, consider translating estimates of relative risk into absolute risk for a meaningful time period |  | Not applicable. |
|  | d) | Consider plots to visualize results (e.g. forest plot, scatterplot of associations between genetic variants and outcome versus between genetic variants and exposure) |  | Supplementary Figure 1-4 are scatterplot of associations between genetic variants and outcome versus between genetic variants and exposure. |
| 12 | **Assessment of assumptions** |  |  |  |
|  | a) | Report the assessment of the validity of the assumptions | 8 | In all maintained SNPs, the F-statistic is greater than 10, indicating sufficient power of correlation between the IVs and the associated bacterial taxon. Consequently, there is no weak instrumental bias in our study (Table S1). |
|  | b) | Report any additional statistics (e.g., assessments of heterogeneity across genetic variants, such as *I^2^*, Q statistic or E-value) | 11 | The Cochran’s Q test outcomes indicated uniformity among the IVs, suggesting homogeneity (Table S3). |
| 13 | **Sensitivity analyses and additional analyses** |  |  |  |
|  | a) | Report any sensitivity analyses to assess the robustness of the main results to violations of the assumptions | 7,8 | To assess the robustness and reliability of our findings regarding the causal association between gut microbiota and GEP-NENs, we conducted a series of sensitivity analyses. (1) Heterogeneity Evaluation: Cochran’s Q test was utilized to examine the heterogeneity among the IVs used in our analysis. (2) Detection of Horizontal Pleiotropy: To identify potential horizontal pleiotropy, where IVs may influence the outcome through pathways other than the exposure of interest, we implemented both MR-Egger intercept and MR-PRESSO global tests. |
|  | b) | Report results from other sensitivity analyses or additional analyses |  | Table S3 |
|  | c) | Report any assessment of direction of causal relationship (e.g., bidirectional MR) |  | Reverse Mendelian randomization analysis and MR-Steiger test |
|  | d) | When relevant, report and compare with estimates from non-MR analyses | 13 | The exploration of the relationship between gut microbiota and GEP-NENs is a burgeoning field, already yielding intriguing findings. Hu et al. analyzed fecal samples from patients with rectal neuroendocrine tumors, unveiling dysbiotic gut microbial and metabolic profiles in these patients (Hu et al., 2022). In another study, Mohamed et al, identified notable differences in bacterial and fungal species between GEP-NEN patients and healthy individuals, highlighting an increase in fungi, particularly Candida, Ascomycota, and Saccharomycetes species (Mohamed et al., 2022). Further contributing to this body of research, Massironi et al, observed bacterial colonization in both intestinal NEN and pancreatic NEN tissues, with a marked increase in bacterial infiltration in pancreatic tumors compared to non-tumoral pancreatic tissue (Pushalkar et al., 2018; Thomas et al., 2018; Massironi et al., 2022). This suggests not only the presence of a pancreatic microbiota in healthy and diseased states but also its potential involvement in tumor pathology. Additionally, Mulders et al, reported a decreased richness and diversity in the gut microbiome of midgut neuroendocrine tumor patients compared to controls, reinforcing the concept of a significant link between gut microbiota alterations and GEP-NENs (Mulders et al., 2024). |
|  | e) | Consider additional plots to visualize results (e.g., leave-one-out analyses) |  | We have provided the results of the Leave-one-out analysis in Supplementary Figure 5-8. |
|  | **DISCUSSION** |  |  |  |
| 14 | **Key results** | Summarize key results with reference to study objectives | 10,11 | We identified 42 taxa of gut microbiota potentially causally associated with GEP-NENs, of which 7 with pancreatic NENs, 8 with colorectal NENs, 11 with small intestinal NENs, and 16 with gastric NENs. After adjusting for false discovery rate (FDR), we found significant causal links: Euryarchaeota with small intestinal NENs, and FamilyXIIIUCG001 with gastric NENs. |
| 15 | **Limitations** | Discuss limitations of the study, taking into account the validity of the IV assumptions, other sources of potential bias, and imprecision. Discuss both direction and magnitude of any potential bias and any efforts to address them | 16 | Our research is not without its limitations. The granularity of the data, confined to the genus level, restricts our ability to examine the causal relationships at the more specific species level, potentially overlooking finer microbial influences on GEP-NENs. Furthermore, the sensitivity analyses and detection of horizontal pleiotropy were constrained by the limited number of genetic variants employed as IVs, with the SNPs used not meeting the conventional GWAS significance threshold (P<5×10^–8^). To mitigate the risk of false positives, we implemented FDR correction. Moreover, while the GWAS meta-analysis for gut microbiota predominantly featured participants of European descent, the potential for population stratification cannot be discounted. Consequently, the applicability of our results to individuals outside this demographic group may be limited. Future MR analyses exploring the causal relationship between gut microbiota and GEP-NENs should consider including a more diverse array of populations, spanning both European and non-European backgrounds, to enhance the generalizability of the findings. Expanding the scope of research in this manner will be crucial for advancing our understanding of GEP-NENs and developing universally effective interventions. |
| 16 | **Interpretation** |  |  |  |
|  | a) | Meaning: Give a cautious overall interpretation of results in the context of their limitations and in comparison with other studies | 13-15 | The exploration of the relationship between gut microbiota and GEP-NENs is a burgeoning field. Several observational studies have confirmed a correlation between gut microbiota dysbiosis and GEP-NENs. We discussed the MR results to the fullest extent and provided a reasonable explanation by comparing them with multiple published studies. |
|  | b) | Mechanism: Discuss underlying biological mechanisms that could drive a potential causal relationship between the investigated exposure and the outcome, and whether the gene-environment equivalence assumption is reasonable. Use causal language carefully, clarifying that IV estimates may provide causal effects only under certain assumptions | 14,15 | Previous studies have confirmed a correlation between gut microbiota dysbiosis and GEP-NENs. Our study found that Euryarchaeota has a protective effect on small intestinal NENs. Euryarchaeota play a pivotal role, engaging in synergistic interactions with bacteria to facilitate the production of short-chain fatty acids(SCFAs) (Lurie-Weinberger and Gophna, 2015). These compounds, particularly propionate and butyrate, are lauded for their cancer-suppressive capabilities within the gastrointestinal tract (Louis et al., 2014), offering a potential mechanistic explanation for the protective effect observed in our study.  Our study also found the genus.FamilyXIIIUCG001 plays a potentially hazardous causal relationship with gastric NENs, however, the exploration of FamilyXIIIUCG001's impact on gastrointestinal diseases, particularly gastric NENs, remains largely uncharted, need for further research into the mechanisms underlying this association. |
|  | c) | Clinical relevance: Discuss whether the results have clinical or public policy relevance, and to what extent they inform effect sizes of possible interventions | 14,15 | Euryarchaeota may serve as probiotics for small intestinal NENs, while the genus FamilyXIIIUCG001 serve as risk factor for gastric NENs, but further research is needed to elucidate the underlying mechanisms. |
| 17 | **Generalizability** | Discuss the generalizability of the study results (a) to other populations, (b) across other exposure periods/timings, and (c) across other levels of exposure | 16 | As the majority of participants in the GWAS data of gut microbiota were of European ancestry, the external validity of our findings to other ethnic groups may be constrained. |
|  | **OTHER INFORMATION** |  |  |  |
| 18 | **Funding** | Describe sources of funding and the role of funders in the present study and, if applicable, sources of funding for the databases and original study or studies on which the present study is based |  | Funding information  This research was supported by the National Natural Science Foundation of China [82104792], Natural Science Foundation of Jiangsu Province [BK20210134], Suzhou Science and Technology Development Plan Project [SKY2023015], Project of National Clinical Research Base of Traditional Chinese Medicine in Jiangsu Province, China [JD2022SZ19], and Suzhou project of diagnosis and treatment technology for clinical key diseases [LCZX202120].  These funding organizations did not participate in the study design, data collection, analysis, interpretation or writing of the manuscript or in the decision to submit the manuscript for publication. |
| 19 | **Data and data sharing** | Provide the data used to perform all analyses or report where and how the data can be accessed, and reference these sources in the article. Provide the statistical code needed to reproduce the results in the article, or report whether the code is publicly accessible and if so, where |  | Generated Statement: Publicly available datasets were analyzed in this study.  The summary data on gut microbiota is from MiBioGen consortium, which can be obtained from the IEU GWAS database (https://gwas.mrcieu.ac.uk/) (GWAS ID: ebi-a-GCST90016908-- ebi-a-GCST90017118).  The summary data on GEP-NENs are from the FinnGen research project.  Pancreatic NENs  (https://r10.finngen.fi/pheno/C3_PANCREAS_NEUROENDOCRINE_EXALLC)  Colorectal NENs  (https://r10.finngen.fi/pheno/C3_COLORECTAL_NEUROENDO_EXALLC)  Small intestinal NENs  (https://r10.finngen.fi/pheno/C3_SMALL_INTESTINE_NEUROENDOCRINE_EXALLC)  Gastric NENs  (https://r10.finngen.fi/pheno/C3_STOMACH_NEUROENDOCRINE_EXALLC) |
| 20 | **Conflicts of Interest** | All authors should declare all potential conflicts of interest |  | The authors declare that the research was conducted in the absence of any commercial or financial relationships that could be construed as a potential conflict of interest. |

This checklist is copyrighted by the Equator Network under the Creative Commons Attribution 3.0 Unported (CC BY 3.0) license.

1. Skrivankova VW, Richmond RC, Woolf BAR, Yarmolinsky J, Davies NM, Swanson SA, et al. Strengthening the Reporting of Observational Studies in Epidemiology using Mendelian Randomization (STROBE-MR) Statement. JAMA. 2021;under review.

2. Skrivankova VW, Richmond RC, Woolf BAR, Davies NM, Swanson SA, VanderWeele TJ, et al. Strengthening the Reporting of Observational Studies in Epidemiology using Mendelian Randomisation (STROBE-MR): Explanation and Elaboration. BMJ. 2021;375:n2233.
