# Supplementary figures and images for: Investigating the causal relationship between gut microbiota and gastroenteropancreatic neuroendocrine neoplasms: a bidirectional Mendelian randomization study
Source: Front Microbiol. 2024 Aug 13;15:1420167. doi: 10.3389/fmicb.2024.1420167 (PMC11347282; doi:10.3389/fmicb.2024.1420167)

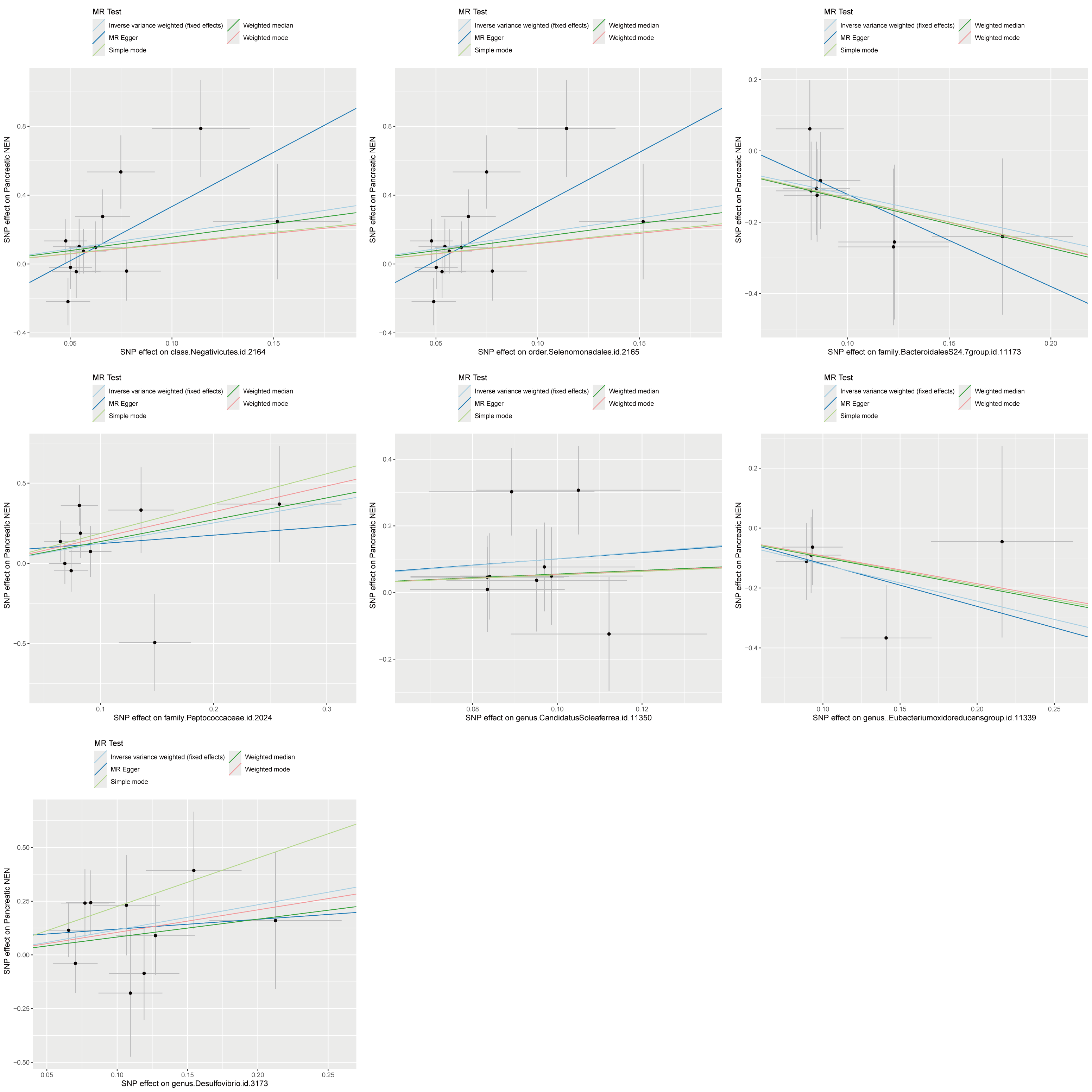

Supplement: Supplementary file 3 [file Image_1.TIF]

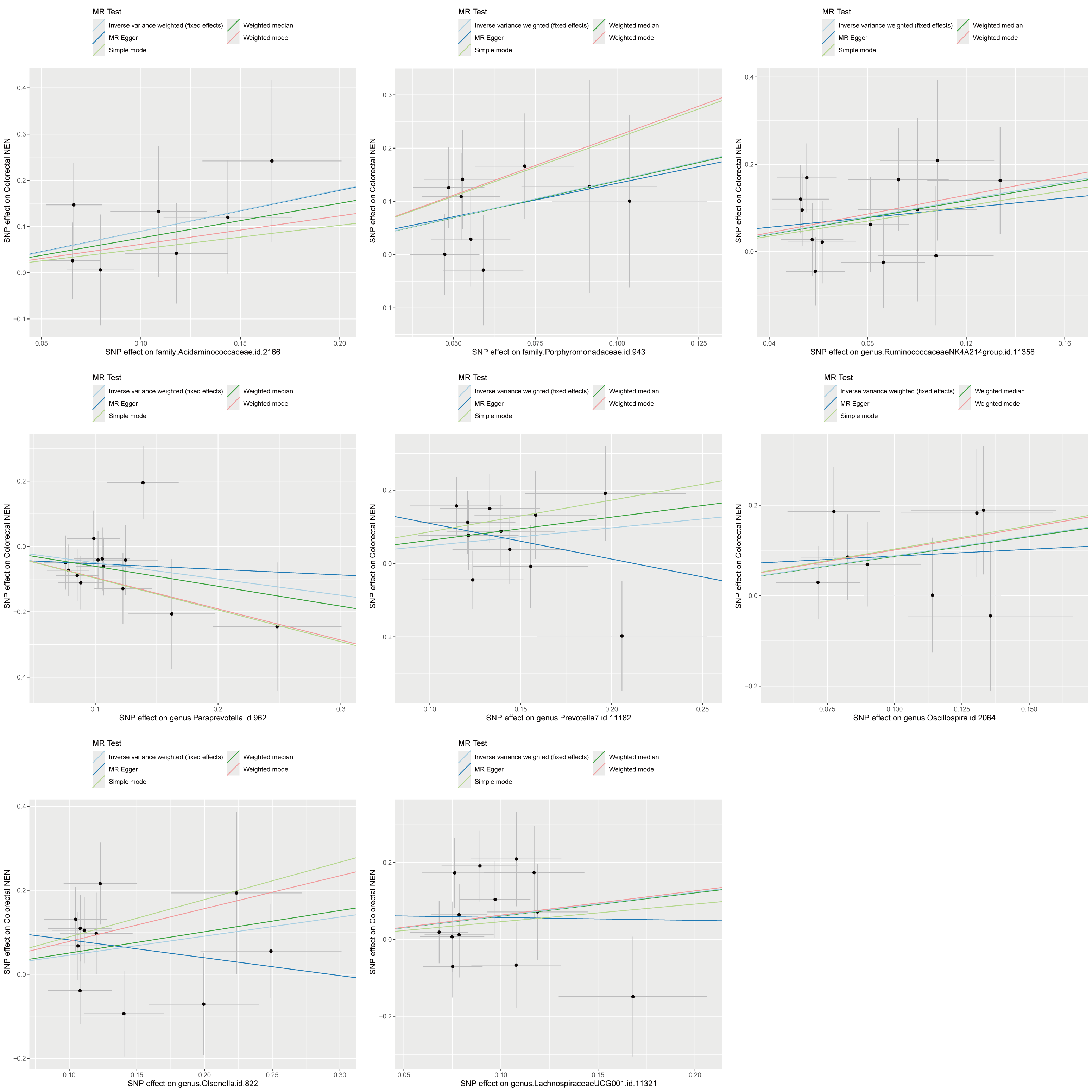

Supplement: Supplementary file 4 [file Image_2.TIF]

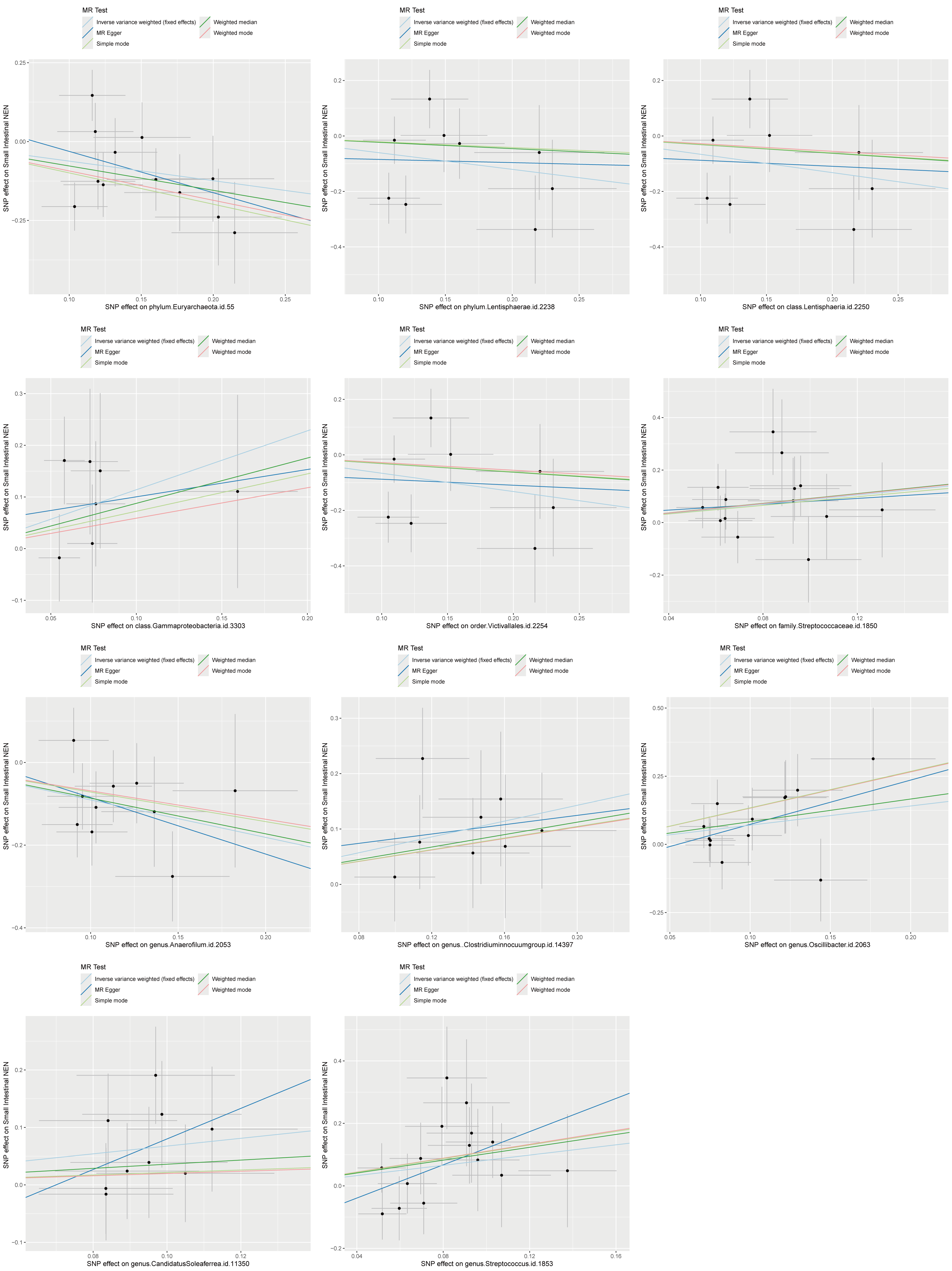

Supplement: Supplementary file 5 [file Image_3.TIF]

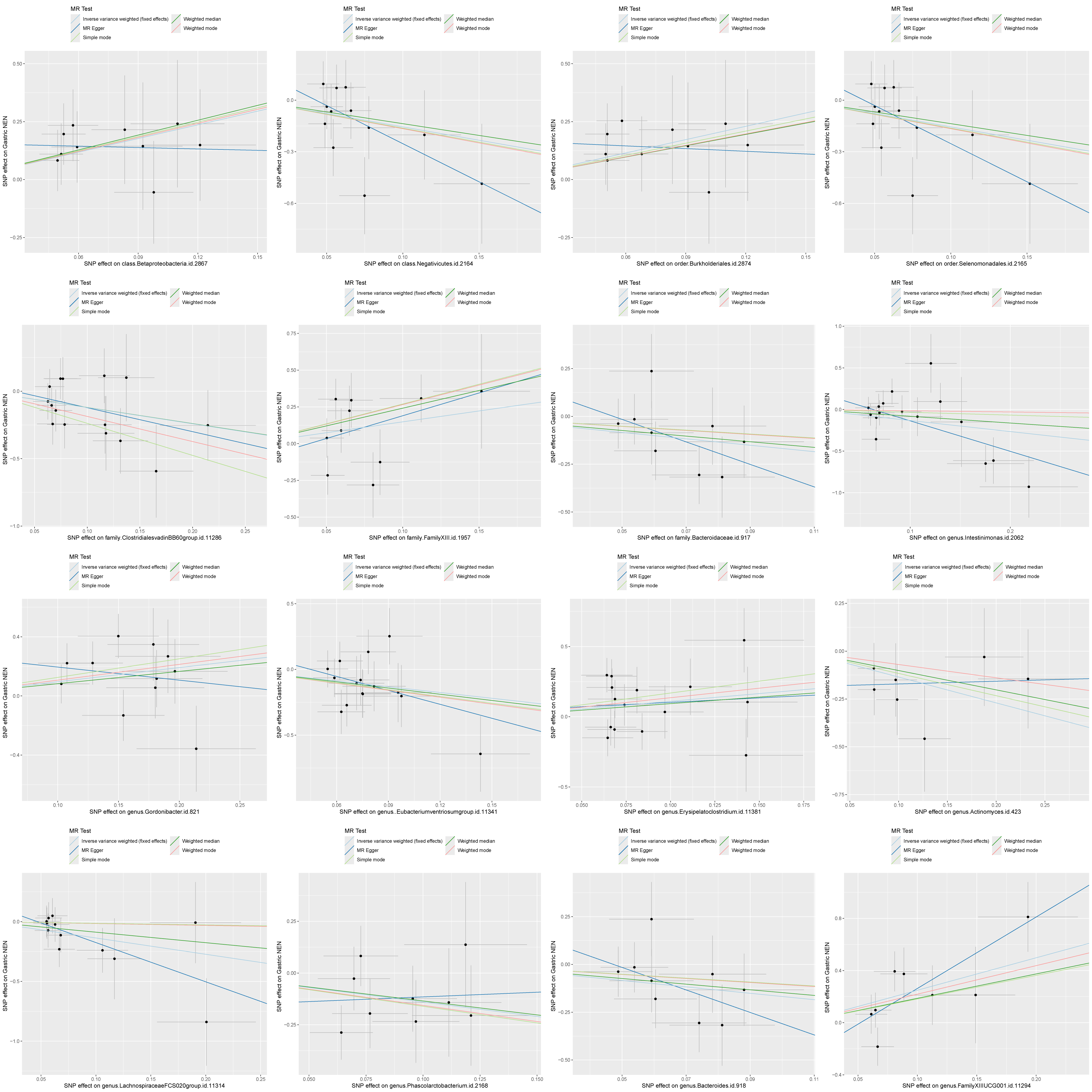

Supplement: Supplementary file 6 [file Image_4.TIF]

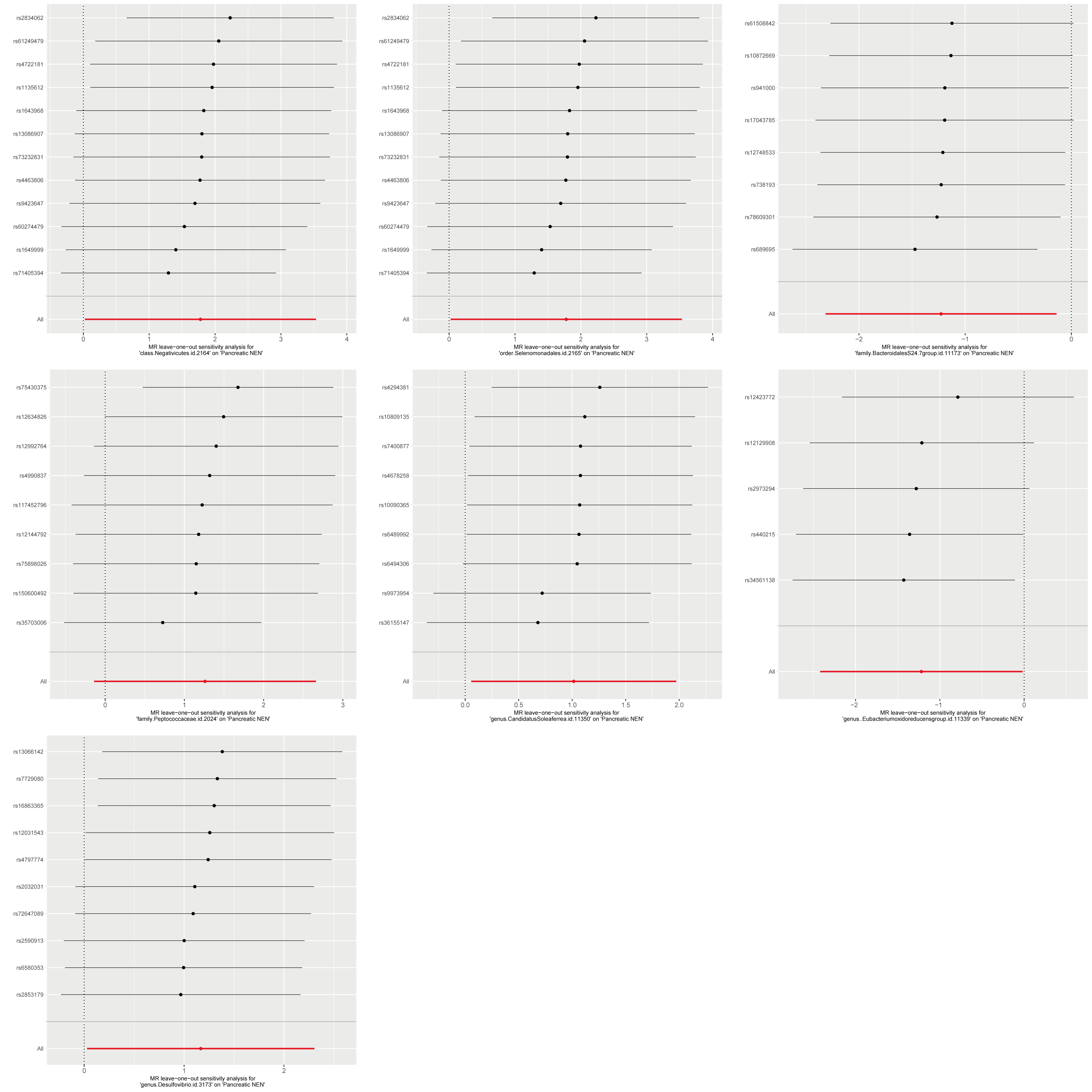

Supplement: Supplementary file 7 [file Image_5.TIF]

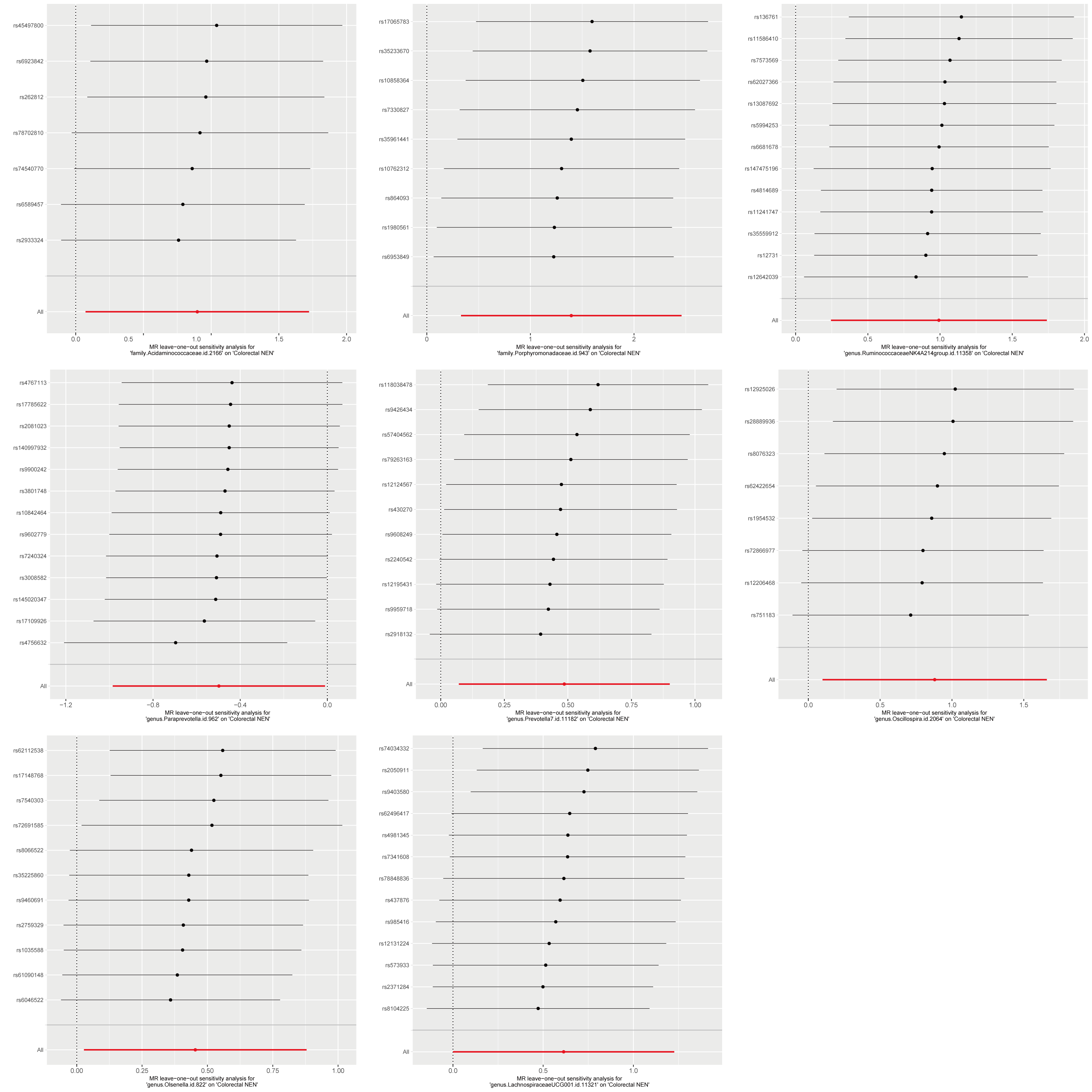

Supplement: Supplementary file 8 [file Image_6.TIF]

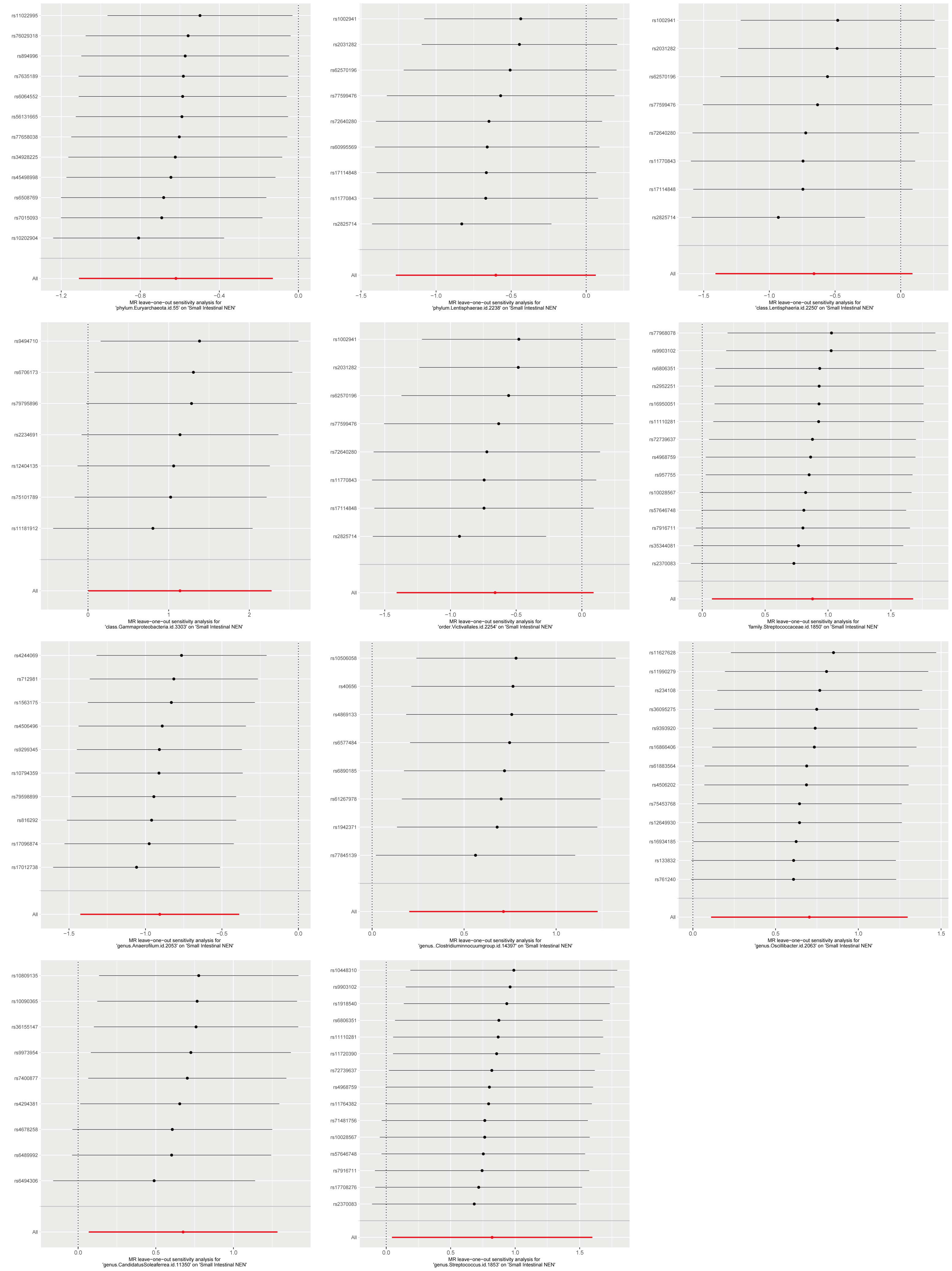

Supplement: Supplementary file 9 [file Image_7.TIF]

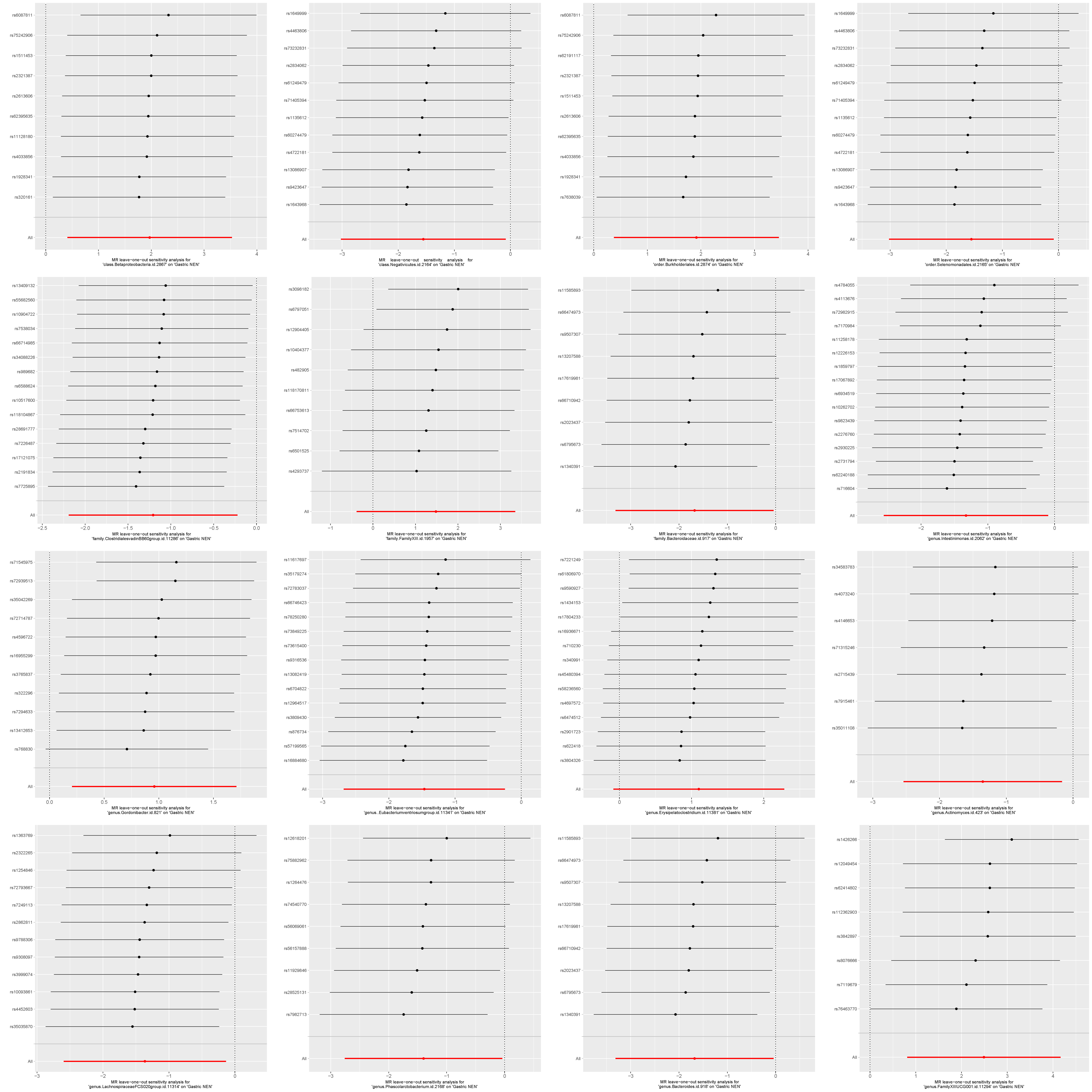

Supplement: Supplementary file 10 [file Image_8.TIF]
